# Short-Term Continuous Glucose Forecasting with Large-Language-Model-Derived Nutrient Estimates from Real-World Chinese Dietary Records

**DOI:** 10.34133/hds.0471

**Published:** 2026-10-28

**Authors:** Leyao Ma, Jie Hao, Lin Yang, Xiaowei Xu, Jiao Li

**Affiliations:** Institute of Medical Information, Chinese Academy of Medical Sciences & Peking Union Medical College, Beijing, China.

## Abstract

**Background:** Accurate short-term glucose forecasting is important for diabetes self-management, yet dietary information remains difficult to quantify and integrate into forecasting models in free-living settings, especially for complex Chinese meals. **Methods:** We investigated whether large language models (LLMs) can convert free-text Chinese dietary records into structured nutritional features for short-term continuous glucose forecasting. Five Chinese-capable LLMs were evaluated as nutrient estimators, and the resulting nutritional features were integrated with continuous glucose monitoring histories in a seasonal-patch transformer for glucose forecasting. Performance was assessed in 2 real-world cohorts (ShanghaiT1DM and ShanghaiT2DM) for both full-period and postprandial glucose forecasting. **Results:** LLM-derived nutrient estimates significantly outperformed semantic embeddings as dietary representations for glucose forecasting (*P* < 0.001). In the primary comparison setting, the best-performing LLM-based model achieved a postprandial 3-h root mean square error (RMSE) of 34.668 mg/dl, reducing the error by 1.967 mg/dl compared to non-LLM settings. In the full-period setting, the optimal LLM-based model achieved an RMSE of 27.035 mg/dl, reducing the error by 0.42 mg/dl relative to non-LLM settings. Across both forecasting tasks, direct nutrient estimation by DeepSeek-v3.1 achieved the best average RMSE. Forecasting gains were positively associated with nutrient-estimation quality, particularly for carbohydrate, dietary fiber, and fat. Architecture ablations further showed that patch-based encoding was the most influential component, with additional contributions from temporal decomposition and side branch design. **Conclusion:** These findings show that LLM-based nutrient estimation provides a practical and physiologically interpretable way to convert free-text dietary records into structured meal-related inputs for glucose forecasting.

## Introduction

Continuous glucose monitoring (CGM) is widely used for self-management in type 1 and type 2 diabetes mellitus (T1DM and T2DM) and has been associated with improved clinical outcomes [1]. By continuously measuring interstitial glucose using minimally invasive subcutaneous sensors, CGM enables individuals to track glucose dynamics outside the clinic. However, CGM readings exhibit a physiological lag relative to blood glucose [2], making continuous glucose forecasting necessary. Accurate short-term glucose forecasting can therefore complement CGM by enabling earlier insulin adjustments and proactive prevention of hypo- and hyperglycemia, ultimately improving daily management and health outcomes [3].

Prior work on continuous glucose forecasting spans data-driven, physiology-based, and hybrid approaches. Recent efforts have largely adopted deep learning models, such as long short-term memory networks and transformer variants, and some studies incorporate exogenous inputs, including physical activity and meal information, to improve predictive performance [4]. In general, forecasting models learn from historical glucose trajectories and may further integrate contextual features, including insulin dosing, exercise, or dietary intake. Incorporating such exogenous information has been shown to improve glucose-forecasting accuracy [5]. Among dietary signals, carbohydrate intake is the most commonly used, given its direct influence on postprandial glucose excursions [6].

However, fully leveraging dietary information remains a major challenge in continuous glucose forecasting. This limitation is partly driven by data availability, as widely used public datasets such as OhioT1DM typically record only carbohydrate intake [7]. Limiting dietary inputs to carbohydrates overlooks other nutritional factors. Fiber, fat, and glycemic index (GI) also influence glucose dynamics and postprandial responses [8]. In real-world settings, dietary records are often unstructured, and obtaining detailed nutrient breakdowns is costly and time-consuming [9]. The challenge is further amplified for Chinese cuisine, where dishes commonly involve complex ingredient combinations and diverse preparation styles that are not well represented in conventional nutrition databases [10]. As a result, prior attempts to model dietary impacts on glucose in Chinese populations have largely relied on expert-curated knowledge rather than practical automated tools [11].

Large language models (LLMs) are increasingly applied in medicine and nutrition. They can extract and structure information from clinical text and have been used for data annotation and downstream predictive modeling [12,13]. For dietary data, LLMs can parse natural-language meal descriptions and generate structured nutrient estimates. Recent studies have evaluated LLMs for macronutrient estimation from meal descriptions and reported estimates comparable to those of professional nutritionists while requiring less time [14]. Early work has also applied LLM-based nutrient estimation to enhance glucose forecasting among virtual patients in an English-language context [15]. The impact of LLM-derived nutrient estimates on forecasting performance remains unclear in real-world cohorts with diverse individuals and cultures.

In this study, we investigated whether LLM-derived nutrient estimates can improve short-term glucose forecasting from real-world Chinese dietary records. We evaluated multiple nutrient-estimation strategies and 5 Chinese-capable LLMs and compared their downstream utility in a seasonal-patch transformer (SPT) forecasting framework. Our contributions are 3-fold. First, we developed a practical workflow for transforming free-text Chinese dietary records into structured nutrient features for CGM forecasting. Second, we systematically compared LLM-derived nutrient estimates with raw semantic meal embeddings and examined the relationship between nutrient-estimation quality and downstream forecasting performance. Third, we performed feature and architecture ablations to clarify which input settings and model components contributed most to forecasting performance.

## Materials and Methods

### Overview of methods

An overview of the proposed workflow is illustrated in Fig. 1. The overall workflow consists of 4 main stages: (a) dataset construction and preprocessing, (b) LLM-based nutrient estimation from Chinese dietary records, (c) CGM forecasting using the proposed SPT, and (d) evaluation of both nutrient-estimation accuracy and forecasting performance.

**Fig. 1.**
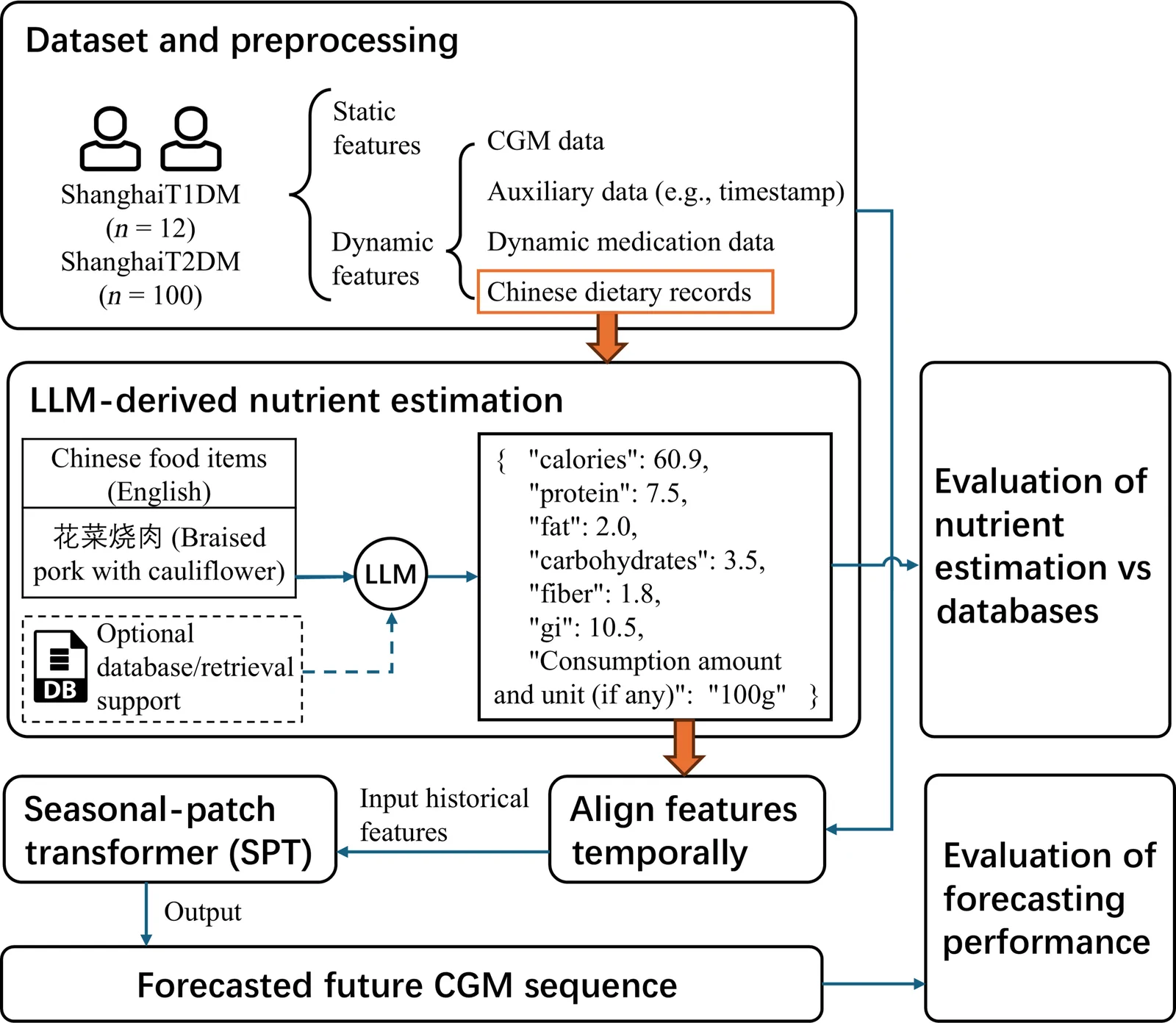
End-to-end workflow for integrating large language model (LLM)-derived nutrient estimation into continuous glucose forecasting. The framework starts with dataset construction, including static and dynamic features such as continuous glucose monitoring (CGM) measurements, auxiliary variables, medication records, and Chinese dietary records. Chinese food items are extracted from dietary records and fed into an LLM to generate structured nutrient estimates with optional database or retrieval support. These features are then temporally aligned and used as historical inputs to the proposed seasonal-patch transformer (SPT) model for forecasting future CGM sequences. The right panel summarizes evaluation from 2 aspects: the accuracy of LLM-derived nutrient estimation (against reference databases) and the performance of CGM prediction. Illustration icons used in this figure were sourced from iSlide’s icon resources (https://www.islide.cc/).

Free-text dietary records were transformed into structured nutritional information using 3 LLM-based strategies, including direct estimation, hybrid LLM–database estimation, and retrieval-augmented generation (RAG). As a comparator, diet word embeddings were also extracted directly from food names. These alternative dietary representations were then incorporated into the proposed SPT model for glucose forecasting.

To evaluate the robustness of performance gains attributable to LLM-derived nutritional features across different settings, we considered multiple feature groups, including medication records and patient-level static features. These features were integrated using the SPT for glucose forecasting under both full-period and postprandial settings. Model evaluation further included statistical analyses of both forecasting performance and nutrient-estimation accuracy.

### Dataset and preprocessing

We used the publicly described ShanghaiT1DM (*n* = 12) and ShanghaiT2DM (*n* = 100) datasets, collected under real-life conditions with CGM sampling by flash glucose monitoring devices (FreeStyle Libre H, Abbott Diabetes Care, Witney, UK) every 15 min over 3 to 14 d per patient [9]. The available variables were organized into static and dynamic components to facilitate downstream modeling.

Specifically, static variables refer to patient-level baseline characteristics, including demographic attributes and initial clinical measurements (e.g., age, body mass index, and baseline laboratory indicators). In contrast, time-dependent information was categorized as dynamic variables. Among these, dynamic medication information denotes time-aligned treatment records synchronized with the CGM sequence, including insulin administration via multiple routes and regimens (e.g., subcutaneous insulin dose, intravenous insulin dose, continuous subcutaneous insulin infusion basal and bolus insulin, and non-insulin hypoglycemic agents).

Dietary information was encoded through 2 methodologically distinct approaches. The first, LLM-derived nutrient features correspond to structured nutritional estimates obtained from free-text meal records. Specifically, Chinese dietary records were parsed using regular expressions to extract food items, quantities, and units, followed by standardization and deduplication. Each food item was then mapped to nutrient compositions (including macronutrients and weighted GI, per 100 g or ml) using an LLM. Macronutrients were aggregated into meal-level nutrient features weighted by intake amounts and GI further weighted by the proportion of carbohydrate contribution. The second approach, diet word embeddings, captures the semantic content of meals. Extracted food names were embedded using the pre-trained model BGE-M3 [16,17] and subsequently aggregated according to consumption quantities to form meal-level representations.

In addition, auxiliary dynamic variables include supporting time-series information such as capillary blood glucose (CBG) measurements and corresponding recording timestamps, which provide complementary signals to CGM data.

Unless otherwise specified, these terms—static features, dynamic medication features, LLM-derived nutrient features (hereafter nutrient features), diet word embeddings, and auxiliary dynamic features—are used consistently throughout the paper to denote the corresponding categories of features to input into the forecasting model. Other preprocessing steps followed standard procedures: categorical variables were one-hot encoded, and continuous variables were normalized using robust scaling based on training set statistics. Detailed preprocessing procedures for all variables are provided in Table S1.

### LLM-derived nutrient estimation

Nutritional feature extraction, or nutrient estimation, was implemented by leveraging LLMs to derive structured nutritional compositions from textual food descriptions. We evaluated 3 LLM-based nutrient-estimation strategies: direct estimation, a hybrid LLM–database approach that only estimates components not covered by the database, and an exploratory novel RAG solution.

Five LLMs were employed to process the Chinese dietary records. These included the closed-source model GPT-5-mini-2025-08-07 [18], along with the open-weight models DeepSeek-v3.1-2025-08-21 [19], Llama-3.1-70B [20], glm-4.5-air [21], and Qwen3-235b-a22b-fp8 [22]. The selection was guided by both task and language considerations. Given the short context and batch-processing demands of nutrient estimation, we prioritized smaller, more efficient models over their larger or thinking-enabled counterparts. Additionally, for this Chinese language task, we favored models developed within China, leveraging their inherent advantages in processing and understanding Chinese text.

We used a chain-of-thought-like prompt strategy inspired by the NutriBench research [15]. Each LLM was instructed to act as a professional nutritionist and to output nutritional information in JavaScript Object Notation (JSON) format. The target fields included calories (kcal), protein (g), fat (g), carbohydrate (g), dietary fiber (g), and weighted GI [11] per 100 g (or ml) of food. The JSON structure’s initial key is designated as a “thinking process step-by-step”. The prompt also required the model to record nonstandard units when present, because purely rule-based parsing of free-text diet logs may miss expressions such as “a slice of bread”. Recording unit information improved the robustness of automated calorie and nutrient aggregation. The full nutrient-estimation prompt is provided in Fig. S1.

We tested the performance of a hybrid LLM-database strategy on all LLMs, prioritizing database exact matches for nutritional information and employing LLM estimates for unmatched data and weighted GI. This approach aligns with the computational resource optimization required for real-world deployment. The nutrition database was constructed from 2 complementary sources: the sixth edition of the Chinese Food Composition (CFC) Table [23,24], providing foundational data for raw ingredients, and our previously established Chinese food dish nutrition database [25] (hereafter dishes database) covering prepared dishes and regional cuisines absent from standard tables.

RAG was included as an exploratory strategy rather than the primary design choice, given prior evidence that retrieval may introduce noise into LLM-based nutrient estimation [15]. However, we explored a novel RAG pipeline based on DeepSeek-v3.1 for food unmatched by the CFC database. For this strategy, the dishes database was retained as an evaluation dataset for the RAG approach, while the CFC database was used as the retrieval source to augment LLM inputs. For food items that can be exactly matched in the CFC database, nutrient values were directly assigned without invoking RAG. For food items not exactly matched in the CFC database, we implemented a 2-stage RAG pipeline. First, candidate dishes were retrieved via jieba-based tokenization [26] and fuzzy matching. An evaluation LLM then assessed whether the retrieved candidates were sufficient. If sufficient, they were directly used for nutrient estimation; otherwise, the food item was decomposed into ingredients, and each ingredient was matched against the database using exact-then-fuzzy retrieval, retaining the top 5 candidates per ingredient. All retrieved candidates were then aggregated and incorporated into the final prompt for nutrient estimation. Figure S2 provides the ingredient-decomposition prompt, and Algorithm S1 summarizes the overall RAG workflow.

### Forecasting task definition

We considered the task of forecasting CGM measurements in patients using a multisource feature fusion time-series framework. Let L denote the sequence length, $F_{d}$ denote the number of dynamic features, and $F_{s}$ denote the number of static features. The objective is to predict the target CGM values at a future horizon of H steps.

The input can include both dynamic and static features:

• Dynamic features $x_{\mathrm{dyn}}\in {\mathbb{R}}^{L\times F_{d}}$ denote the structured dynamic inputs, composed of the CGM sequence $x_{\mathrm{CGM}}$, nutritional features $x_{\mathrm{nut}}$ (macronutrients and weighted GI), dynamic medication features $x_{\mathrm{med}}$, and auxiliary time-varying covariates $x_{\mathrm{aux}}$ (CBG, recording time), where $F_{d}=1+F_{\mathrm{nut}}+F_{\mathrm{aux}}+F_{\mathrm{med}}$.
• Diet word embeddings $x_{\mathrm{emb}}\in {\mathbb{R}}^{L\times F_{e}}$ denote the optional meal-text embedding sequence, where $F_{e}$ is the embedding dimension.
• Static features $x_{\text{stat}}\in {\mathbb{R}}^{F_{s}}$ include patient-specific attributes such as age, body mass index, and baseline clinical measurements.
• Dynamic missingness masks $M_{\mathrm{dyn}}\in {\left\{0,\;1\right\}}^{L\times F_{d}}$ indicate observed (1) and missing (0) entries in the structured dynamic features $x_{\mathrm{dyn}}$.
• Diet observation masks $M_{\mathrm{emb}}\in {\left\{0,\;1\right\}}^{L}$ indicate whether diet embedding is available at each time step.
• Static missingness masks $M_{\text{stat}}\in {\left\{0,\;1\right\}}^{F_{s}}$ indicate observed (1) and missing (0) entries in the static features.

The forecasting target is the future CGM measurements:

$$y=\left[{\mathrm{CGM}}_{t+1},\ldots ,{\mathrm{CGM}}_{t+H}\right]\in {\mathbb{R}}^{H}.$$ (1)

Given the historical structured dynamic input $x_{\mathrm{dyn}}$, together with optional static covariates $x_{\text{stat}}$ and optional diet-embedding inputs $x_{\mathrm{emb}}$, the goal is to learn a mapping

$$f:\left(x_{\mathrm{dyn}},M_{\mathrm{dyn}},x_{\mathrm{stat}},M_{\mathrm{stat}},x_{\mathrm{emb}},M_{\mathrm{emb}}\right)\mapsto y.$$ (2)

The model was trained to minimize the mean squared error between predicted and true CGM measurements:

$$L_{\mathrm{MSE}}=\frac{\sum_{h=1}^{H}M_{i,h}^{\mathrm{CGM}}{\left({\hat{y}}_{i,h}-y_{i,h}\right)}^{2}}{\sum_{i,h}M_{i,h}^{\mathrm{CGM}}},$$ (3)

where $M^{\mathrm{CGM}}\in {\left\{0,1\right\}}^{H}$ indicates the missingness mask of CGM points.

### Seasonal-patch transformer

The SPT forecasting model includes seasonal decomposition [27], patched input [28], and multiple branches for equitable comparison of different feature configurations, specifically with and without static features, diet embeddings, and nutrition estimation (Fig. 2).

**Fig. 2.**
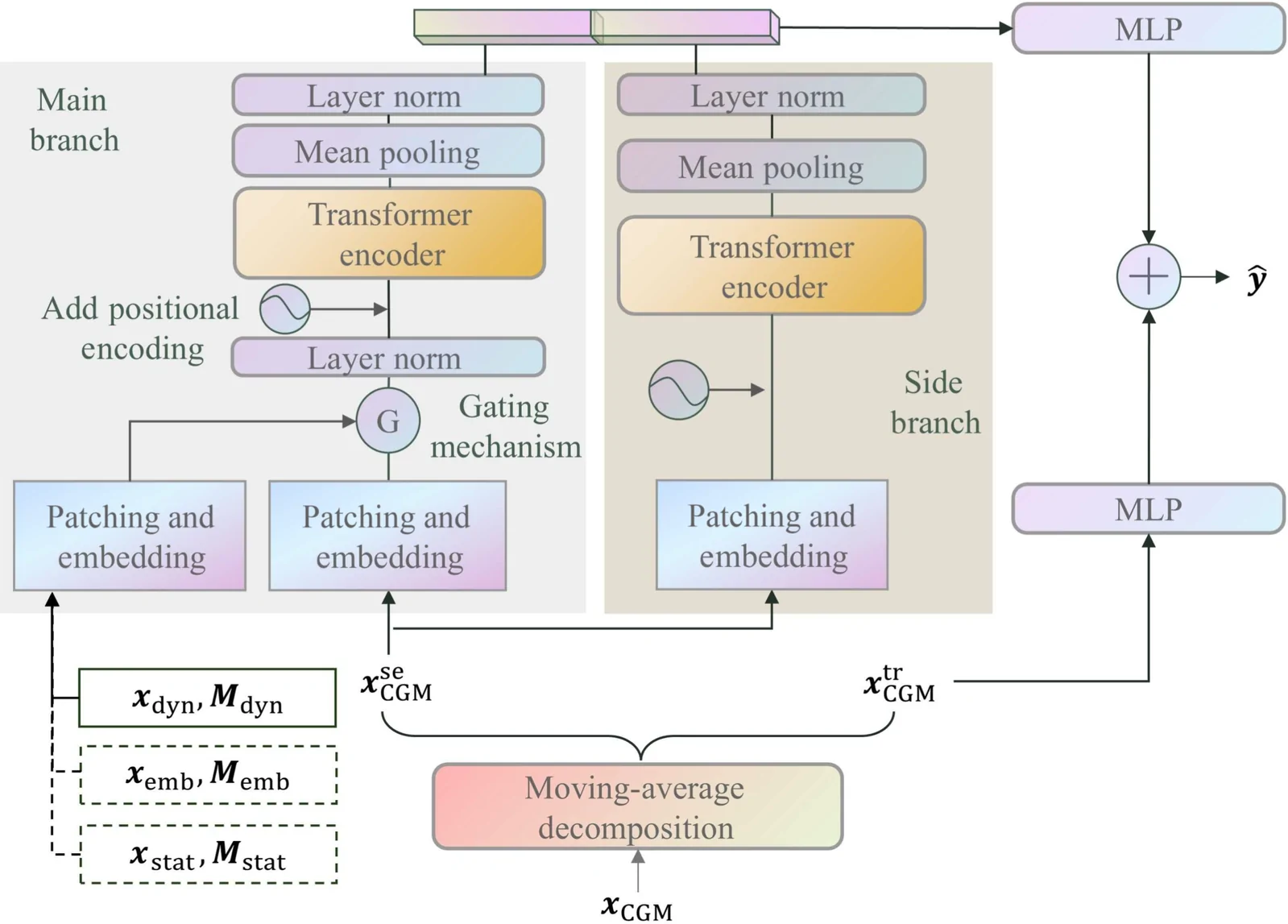
Architecture of the forecasting model. The model takes $x_{\mathrm{CGM}}$ as the primary input, which is decomposed into $x_{\mathrm{CGM}}^{\mathrm{se}}$ and $x_{\mathrm{CGM}}^{\mathrm{tr}}$ by a moving-average decomposition. The 2-branch design and gating mechanism allow selectively incorporating additional features, such that diet word embeddings $x_{\mathrm{emb}}$ and static features $x_{\text{stat}}$ can be omitted. The structured dynamic inputs $x_{\mathrm{dyn}}$ consist of the CGM sequence $x_{\mathrm{CGM}}$, nutritional features $x_{\mathrm{nut}}$, dynamic medication features $x_{\mathrm{med}}$, and auxiliary time-varying covariates $x_{\mathrm{aux}}$, with some feature groups optionally included or excluded depending on the configuration. Some graphical elements in this figure were adapted from ML Visuals, authored by Elvis Saravia and available at https://github.com/dair-ai/ml-visuals.

The proposed SPT decomposes only the historical CGM channel that is part of $x_{\mathrm{dyn}}\in {\mathbb{R}}^{L\times F_{d}}$. Let $x_{\mathrm{CGM}}\in {\mathbb{R}}^{L}$ denote the historical CGM sequence. A moving-average decomposition with kernel size *k* is applied to obtain trend and seasonal components to separate slower background glucose drift from shorter-term fluctuations:

$$x_{\mathrm{CGM}}^{\mathrm{tr}}={\mathrm{MA}}_{k}\left(x_{\mathrm{CGM}}\right),\;x_{\mathrm{CGM}}^{\mathrm{se}}=x_{\mathrm{CGM}}-x_{\mathrm{CGM}}^{\mathrm{tr}}.$$ (4)

The seasonal component is then processed by 2 parallel transformer encoders: the main branch for multimodal feature fusion and the side branch focusing solely on the target’s seasonal history. This parallel design enables a direct comparison of various feature settings.

Following the patching idea of PatchTST, the observation window is divided into $N_{}=\frac{L}{P}$ nonoverlapping patches of length P. Omitting the batch index for clarity, let $I_{i}=\left\{(i-1)P+1,\ldots ,iP\right\}$ denote the temporal index set of patches. Unlike the channel-independent patching in the original PatchTST [28], the present model vectorizes each multivariate patch before linear projection, where $\mathrm{vec}\left(\cdot \right)$ flattens a patch matrix along its temporal and feature dimensions into a single vector while preserving the within-patch temporal order:

$$\begin{array}{c} p_{i}^{\mathrm{se}}=x_{\mathrm{CGM},I_{i}}^{\mathrm{se}}\in {\mathbb{R}}^{P},\\ p_{i}^{\mathrm{dyn}}=\mathrm{vec}\left(x_{\mathrm{dyn},I_{i}}\right)\in {\mathbb{R}}^{P\times F_{d}},\\ p_{i}^{\mathrm{emb}}=\mathrm{vec}\left(x_{\mathrm{emb},I_{i}}\right)\in {\mathbb{R}}^{P\times F_{e}}. \end{array}$$ (5)

This design allows each patch token to jointly encode short-range temporal patterns and cross-variable interactions within the same local window, which is more suitable for CGM forecasting with heterogeneous covariates such as nutrient features, capillary glucose, and time-related information.

The side branch models the seasonal CGM history alone. Each seasonal patch is linearly embedded, augmented with positional encoding, and processed by a transformer encoder:

$$\begin{array}{c} r^{\text{side}}=\mathrm{LN}\;\left(\text{MeanPool}\;\left({\text{Transformer}}_{\text{side}}\;\left(\mathrm{PE}\;\left(W_{\text{side}}p_{i}^{\mathrm{se}}\right)\right)\right)\right), \end{array}$$ (6)

where $\mathrm{LN}\left(\cdot \right)$ denotes layer normalization and PE denotes position encoding.

The target, structured dynamic, and diet-word-derived patch embeddings, together with the shared static embedding, are written as

$$\begin{array}{c} e_{i}^{\mathrm{se}}=\phi_{\mathrm{se}}\;\left(p_{i}^{\mathrm{se}}\right),\\ \;e_{i}^{\mathrm{dyn}}=\phi_{\mathrm{dyn},\mathrm{val}}\;\left(p_{i}^{\mathrm{dyn}}\right)+\phi_{\mathrm{dyn},\mathrm{mask}}\;\left(m_{i}^{\mathrm{dyn}}\right),\\ \;e_{i}^{\mathrm{emb}}=\phi_{\mathrm{emb},\mathrm{val}}\;\left(p_{i}^{\mathrm{emb}}\right)+\phi_{\mathrm{emb},\mathrm{mask}}\;\left(m_{i}^{\mathrm{emb}}\right),\\ \;e^{\mathrm{stat}}=\phi_{\mathrm{stat},\mathrm{val}}\;\left(x_{\mathrm{stat}}\right)+\phi_{\mathrm{stat},\mathrm{mask}}\;\left(M_{\mathrm{stat}}\right) \end{array}$$ (7)

where $\phi_{*}\left(\cdot \right)$ denotes an embedding function implemented as a shallow multilayer perceptron (MLP), $m_{i}^{\mathrm{dyn}}$ is the vectorized patch-level mask derived from $M_{\mathrm{dyn}}$, $m_{i}^{\mathrm{emb}}$ is the patch-level diet-availability indicator, and $e^{\text{stat}}$ is shared across patches. The fusion steps of the patch embeddings share the same gated residual form,

$$G\left(a,\;b\right)=a+\sigma \left(W\left[a;b\right]\right)⊙b$$ (8)

such that

$$h_{i}=G\left(e_{i}^{\mathrm{se}},\;e_{i}^{\mathrm{dyn}}\right)+G\left(e_{i}^{\mathrm{se}},\;e_{i}^{\mathrm{emb}}\right)+G\left(e_{i}^{\mathrm{se}},e^{\mathrm{stat}}\right).$$ (9)

After layer normalization and positional encoding, the patch sequence is passed through the main transformer encoder and mean-pooled:

$$r^{\text{main}}=\mathrm{LN}\;\left(\text{MeanPool}\;\left({\text{Transformer}}_{\text{main}}\;\left(\mathrm{PE}\;\left(\mathrm{LN}\;\left(h\right)\right)\right)\right)\right)$$ (10)

The final prediction combines a seasonal forecast from the fused representation and a trend forecast from the historical trend component. The seasonal forecast is obtained by concatenating the main and side representations, followed by linear fusion and prediction:

$${\hat{y}}^{\mathrm{se}}={\mathrm{MLP}}_{\mathrm{se}}\left(r^{\mathrm{main}},r^{\mathrm{side}}\right)$$ (11)

$${\hat{y}}^{\mathrm{tr}}={\mathrm{MLP}}_{\mathrm{tr}}\left(x_{\mathrm{CGM}}^{\mathrm{tr}}\right)$$ (12)

$$\hat{y}={\hat{y}}^{\mathrm{se}}+{\hat{y}}^{\mathrm{tr}}.$$ (13)

### Evaluation

We evaluated 2 forecasting tasks: full-period glucose forecasting and postprandial glucose forecasting. For full-period forecasting, we used a rolling-window scheme. For postprandial glucose forecasting, we selected data segments where a meal event occurred within the observation window and the forecasting window started within 30 min (2 data points) following the last meal event. The evaluation metrics include root mean square error (RMSE), mean absolute error (MAE), mean absolute relative difference (MARD), and $R^{2}$ between predicted and true glucose values over the 12-point horizon, excluding missing CGM points.

Among the main experiments, the options of whether to use static features, LLM for nutrient estimation, and diet embeddings were controlled. The baseline model used only recording time, CBG, and historical CGM data. Unless otherwise specified, all other experiments used recording time, CBG, historical CGM data, and dynamic medication features.

Next, we conducted a comprehensive analysis of the nutrient-estimation results, including calculations of RMSE against database values, as well as correlation analyses. The databases lack GI data, so only macronutrients were included in the evaluation. We calculated the Spearman correlation coefficient between the RMSE of the LLMs’ nutrient estimates compared to the databases and the average RMSE of the LLM-based glucose forecasting. This aims to uncover the relationship between the accuracy of nutrient estimation and the capability for blood glucose prediction.

To evaluate the contribution of LLM-derived features, dynamic medication features, and the main components of the forecasting model, we performed both feature ablation and architectural ablation studies. All ablation experiments were conducted on the dataset constructed using DeepSeek-v3.1 direct nutrient estimation, as this setting achieved the best average performance in the main experiments. We first conducted a feature ablation on dynamic medication features by comparing the same forecasting backbone with and without medication-related dynamic inputs. Since the model performed better without medication information, all subsequent architectural ablations were conducted under the no-medication setting. Under this unified setting, all models used recording time, CBG, and historical CGM data as inputs, while one architectural component was removed at a time, including the temporal decomposition module, patch-based representation, and side branch.

Unless otherwise specified, model development and evaluation were conducted on the combined ShanghaiT1DM and ShanghaiT2DM cohorts to enhance generalizability by incorporating more diverse real-world glucose dynamics and behavioral patterns. Cohort-specific analyses supporting the rationale for the pooled training strategy are provided in Sections S1.5 and S2.3.

For statistical analysis, 2-group comparisons on RMSE were performed using Welch’s *t* test or the independent-samples *t* test according to the results of Levene’s test for equality of variances. Differences among multiple experimental groups were assessed using the Kruskal–Wallis test. Fisher’s exact test was used to compare the medication usage of 2 patient cohorts. Significance was determined by *P* < 0.05.

### Experiment settings

The dataset was split into patient-level training, validation, and test sets (7:1:2 ratio) with stratification by patient type (T1DM and T2DM). Each experiment was repeated 10 times on the same dataset division to eliminate random errors. For each patient, we slid a 48-point observation window and forecasted the next 12 points (3 h). The full-period glucose-forecasting task consisted of 120,810 input–output pairs, while the postprandial glucose-forecasting task included 6,631 pairs.

The temperature value of LLMs was set to 1 as recommended for most tasks. There were 1,926 unique food items, among which 94 types were precisely matched in the CFC Standard database, and 383 types were precisely matched in the dish nutrition database.

Training of the forecasting models was conducted using the Muon optimizer with Adam [29]. The Muon optimizer was applied to all parameters with dimensions greater than or equal to 2 (with a decay rate of 0.01 and a peak learning rate of 0.001) because it is designed specifically for matrix multiplication, while Adam was used for parameters with dimensions less than 2 (with a decay rate of 0.01 and a peak learning rate of 0.0005).

The kernel size of moving-average decomposition was 3. The patch length was 4. The hidden layer dimension of the forecasting models was 128. Each transformer encoder inside consisted of 6 encoder layers with 8 attention heads. We trained the forecasting models with a batch size of 1,024 for a maximum of 200 epochs, employing a 10-epoch early stopping strategy. The models were developed using Python 3.12 and Torch. All experiments were carried out on NVIDIA Tesla V100 with 32 GB of memory.

## Results

### Forecasting results

Firstly, we examined the overall impact of different feature settings on the forecasting model. Figure 3 illustrates the model performance across feature combinations, highlighting the influence of static features and diet word embeddings under LLM and non-LLM settings. LLM-estimated macronutrients generally led to lower RMSEs. However, the addition of LLM-derived nutritional features surpassed the baseline models only when static features and diet word embeddings were excluded. The models that used only LLM-derived nutritional features were significantly better than the models that used only diet word embeddings (*P* < 0.001 in both tasks). These results suggested that static features and diet word embeddings introduced excessive noise rather than useful information. Without LLM-derived nutrient features, the inclusion of dynamic medication features resulted in poorer postprandial forecasting performance than the baseline model. The results of all experimental combinations are presented in Table S2.

**Fig. 3.**
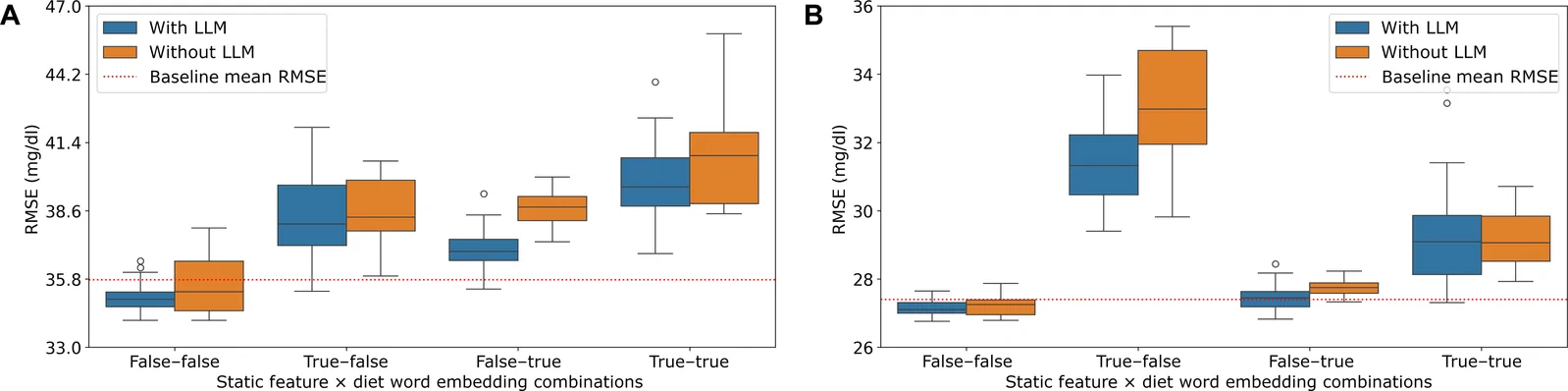
Comparison of glucose-forecasting performance under different feature input combinations with and without large language model (LLM)-based nutrient estimation. Panel (A) shows postprandial glucose-forecasting performance, and panel (B) shows full-period glucose-forecasting performance. The combinations (*X*-axis) are defined by the inclusion or exclusion of patient-level static features and diet word embeddings. For instance, “True–false” indicates the inclusion of static features but the exclusion of diet word embeddings. The blue boxes indicate models with LLM-derived nutrient estimates, while orange boxes represent those without LLM. The dashed horizontal line indicates the mean root mean square error (RMSE) of the baseline model using recording time, capillary blood glucose (CBG), and historical glucose data only. Lower RMSE values indicate better performance.

The models that did not incorporate static features or diet word embeddings performed the best. Therefore, subsequent analyses focused on this group (Table 1). For postprandial CGM forecasting, the models incorporating nutritional estimation performed significantly better than the w/o model (*P* < 0.001) and the baseline model (*P* = 0.003). Among these settings, the direct-estimation method by DeepSeek-v3.1 achieved the greatest number of top-ranked results across the evaluated metrics. It presented an improvement of 1.967 mg/dl compared to the counterpart without LLM-estimated nutrients (mean RMSE = 34.668 mg/dl vs 36.635 mg/dl).

**Table 1.** Average forecasting performance across model settings without static features and diet word embeddings. Note: w/o denotes without, db indicates integration with the nutrition databases, and RAG indicates the use of the RAG technique. ↑ indicates higher is better, and ↓ indicates lower is better. Bold marks the best-performing model for each metric, and underlining marks the second best in the metric. Models achieving the most top 1 metrics per task are highlighted in bold.

Task	Setting	RMSE (mg/dl)↓	MAE (mg/dl)↓	MARD (%)↓	*R*^2^↑
Postprandial	Baseline	35.773	27.267	17.859	0.417
	w/o LLM	36.635	27.773	18.355	0.389
	**DeepSeek-v3.1**	**34.668**	**26.146**	17.582	**0.453**
	DeepSeek-v3.1_db	34.823	26.498	**17.485**	0.448
	DeepSeek-v3.1_RAG	34.862	26.463	17.486	0.446
	GLM4.5	35.144	26.740	17.611	0.439
	GLM4.5_db	35.091	26.623	17.612	0.440
	GPT-5-mini	34.979	26.533	17.581	0.443
	GPT-5-mini_db	34.854	26.426	17.612	0.447
	Llama-3.1	35.119	26.590	17.625	0.438
	Llama-3.1_db	34.718	26.148	17.686	0.452
	Qwen3	35.283	26.700	17.725	0.434
	Qwen3_db	34.845	26.330	17.615	0.447
Full-period	Baseline	27.401	18.807	14.191	0.624
	w/o LLM	27.454	18.800	14.164	0.622
	DeepSeek-v3.1	27.112	18.585	14.017	0.632
	DeepSeek-v3.1_db	27.148	18.530	13.899	0.631
	**DeepSeek-v3.1_RAG**	**27.035**	18.497	**13.843**	**0.634**
	GLM4.5	27.079	18.535	13.930	0.632
	GLM4.5_db	27.125	18.563	13.943	0.631
	GPT-5-mini	27.233	18.553	14.115	0.628
	GPT-5-mini_db	27.084	**18.487**	13.977	0.632
	Llama-3.1	27.118	18.489	13.918	0.631
	Llama-3.1_db	27.163	18.643	13.937	0.630
	Qwen3	27.240	18.654	13.943	0.628
	Qwen3_db	27.097	18.514	13.924	0.632

RAG, retrieval-augmented generation; RMSE, root mean square error; MAE, mean absolute error; MARD, mean absolute relative difference; LLM, large language model

For full-period forecasting, the baseline model achieved the highest mean RMSE (Table 1). The absolute advantage of the nutrient-estimation strategy was relatively smaller than that of the former task, but it was still statistically significant (*P* = 0.001). Within this comparison group, DeepSeek-v3.1_RAG achieved the greatest number of top-ranked results across the evaluated metrics, ranking first in RMSE, MARD, and *R*^2^. Relative to the baseline model, it lowered RMSE from 27.401 to 27.035 mg/dl.

Incorporating external databases sometimes yielded further improvement. For example, Qwen3 with database achieved better postprandial forecasting than the counterpart without database (mean RMSE = 34.845 mg/dl vs 35.283 mg/dl). However, the test comparing the hybrid LLM–database strategy with the direct-estimation strategy for all 5 LLMs did not reveal any significant differences.

In order to examine our exploration of the RAG method, we conducted a Kruskal–Wallis test on the 3 experiments (direct estimation, hybrid LLM–database, and RAG) of the DeepSeek-v3.1 group and found no significant differences, whether in the postprandial task or the full-period forecasting task.

### Nutrient-estimation analyses

We calculated the Spearman correlation coefficient between the RMSE of the LLMs’ nutrient estimates compared to the databases and the average RMSE of the LLM-based glucose forecasting. This part of the analysis was based on experiments that utilized medication data but did not incorporate static and diet word embeddings. To understand the differences among various estimation methods, the methods were ranked by the average RMSE across the 2 tasks (Fig. 4). DeepSeek-v3.1 performed the best, followed by DeepSeek-v3.1_RAG, GPT-5-mini, GLM4.5, Llama-3.1, and Qwen3.

**Fig. 4.**
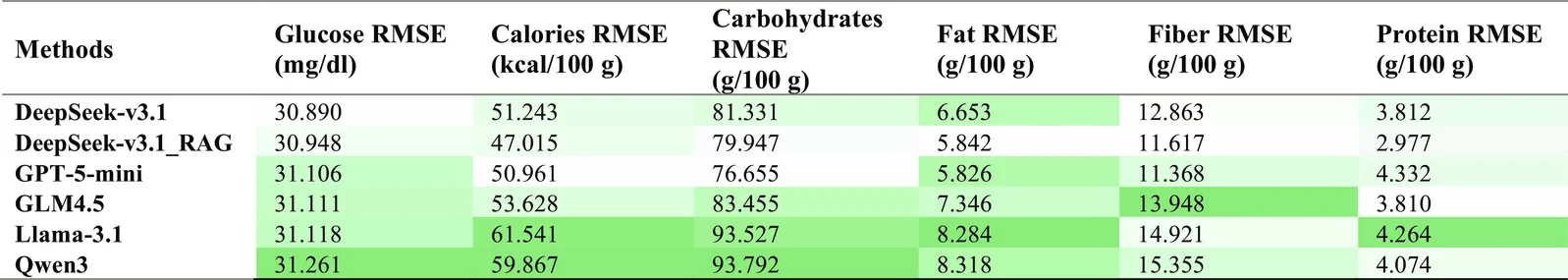
Performance comparison of different large language models (LLMs) across forecasting and nutrition metrics. Note: Mean glucose root mean square error (RMSE) is reported for both full-period and postprandial glucose forecasting. Cell shading indicates the within-column ranking of values.

Although LLM-based nutrient estimations improved CGM forecasting, they still exhibited biases compared to database values. These biases were quantified using precisely matched food items from our mixed database (Fig. 4), which included both raw ingredients and composite dishes. DeepSeek-v3.1, GLM4.5, and GPT-5-mini each had 477 matches, while Llama-3.1 and Qwen3 showed slightly lower counts of 460 and 455, respectively, due to some outputs lacking unit annotations that were consequently excluded. Notably, the RAG’s 383 entries consisted entirely of items not found in the CFC database for fair comparison. Correlation analysis between the biases and forecasting rankings revealed the following order of association: carbohydrates (*ρ* = 0.77), calories (*ρ* = 0.77), fiber (*ρ* = 0.66), fat (*ρ* = 0.49), and protein (*ρ* = 0.09).

### Ablation study

We performed the ablation study in 2 stages. In the main experiments, adding dynamic medication features to the baseline setting did not improve forecasting performance, whereas the combination of LLM-derived nutrient features and dynamic medication features still outperformed the corresponding non-LLM setting. This pattern suggested that the dominant gain came from the LLM-derived dietary representation rather than from medication inputs alone. We therefore first examined whether dynamic medication features provided additional benefit on top of the best-performing DeepSeek-v3.1 direct-estimation setting. Next, we evaluated the architectural variants under that best input configuration. This design allowed us to disentangle the contribution of dynamic medication features from the contribution of the proposed SPT components.

#### Feature-level ablation on dynamic medication features

Removing dynamic medication features led to a slight numerical improvement in both evaluation settings (Table 2). RMSE decreased from 27.112 to 26.963 mg/dl in the full-period analysis and from 34.668 to 34.481 mg/dl in the postprandial analysis, with both configurations remaining significantly better than the main experimental baseline (*P* < 0.001). However, neither improvement reached statistical significance (*P* = 0.0840 and *P* = 0.2219, respectively). These findings suggest that dynamic medication features did not provide a stable incremental benefit under pooled T1DM/T2DM training.

**Table 2.** Feature ablation on dynamic medication features. Note: *P* values are obtained by comparison within one task. “*” for *P* < 0.05 and ns = not significant.

Task	Variant	RMSE (mg/dl)↓ (*P*)	MAE (mg/dl)↓ (*P*)	MARD (%)↓ (*P*)	*R*^2^↑ (*P*)
Full-period	SPT with dynamic medication features	27.112 (reference)	18.585 (reference)	14.017 (reference)	0.632 (reference)
	SPT w/o dynamic medication features	26.963 (0.084 ns)	18.467 (0.156 ns)	13.825 (0.015 *)	0.635 (0.084 ns)
Postprandial	SPT with dynamic medication features	34.668 (reference)	26.146 (reference)	17.582 (reference)	0.453 (reference)
	SPT w/o dynamic medication features	34.481 (0.222 ns)	26.150 (0.986 ns)	17.460 (0.245 ns)	0.459 (0.220 ns)

RMSE, root mean square error; MAE, mean absolute error; MARD, mean absolute relative difference; SPT, seasonal-patch transformer

One possible explanation is the substantial heterogeneity in medication regimens between the 2 cohorts (see Section S2.2), which introduces distributional inconsistency and increases the difficulty of learning a unified representation of medication effects across cohorts. Any insulin exposure was observed in all T1DM patients but in only 63.2% of T2DM patients (12/12 vs 60/100, *P* = 0.0040), whereas non-insulin hypoglycemic agents were substantially more prevalent in T2DM than in T1DM (84/100 vs 6/12, *P* = 0.0124).

#### Architectural ablation

We adopted SPT without dynamic medication features as the reference setting for its better performance. We compared the full model against 3 variants: (a) w/o Decomp, directly processing raw CGM without the decomposition module and its associated trend-forecasting path; (b) w/o Patch, using per-step embeddings instead of patch-wise representation; and (c) w/o Side Branch, removing the side branch that only processes CGM data. Table 3 presents the results.

**Table 3.** Architectural ablations relative to the full model. Note: Positive Δ values for RMSE (mg/dl), MAE (mg/dl), and MARD (%) indicate worse performance than the reference model, whereas negative Δ values for *R*^2^ indicate reduced explained variance. *P* values were obtained by comparing each ablation variant against the corresponding SPT without dynamic medication features under the same evaluation setting. “***” for *P* < 0.001; “**” for *P* < 0.01; “*” for *P* < 0.05; ns = not significant.

Setting	Variant	ΔRMSE (*P*)	ΔMAE (*P*)	ΔMARD (*P*)	Δ*R*^2^ (*P*)
Full-period	w/o Decomp	+0.534 (0.004 **)	+0.731 (0.004 **)	+0.522 (0.002 **)	−0.015 (0.004 **)
	w/o Patch	+0.520 (0.004 **)	+0.315 (0.014 *)	+0.396 (0.002 **)	−0.014 (0.004 **)
	w/o Side Branch	+0.233 (0.084 ns)	+0.140 (0.160 ns)	+0.219 (0.084 ns)	−0.006 (0.084 ns)
Postprandial	w/o Decomp	+0.608 (0.027 *)	+0.461 (0.131 ns)	+0.244 (0.160 ns)	−0.019 (0.027 *)
	w/o Patch	+1.103 (0.002 **)	+0.863 (0.006 **)	+0.740 (0.002 **)	−0.035 (0.002 **)
	w/o Side Branch	+0.479 (0.037 *)	+0.310 (0.160 ns)	+0.231 (0.084 ns)	−0.015 (0.037 *)

RMSE, root mean square error; MAE, mean absolute error; MARD, mean absolute relative difference; SPT, seasonal-patch transformer

Removing any of the 3 key modules reduced performance relative to the reference model (Table 3). Among them, patch-based encoding had the largest and most consistent effect. Removing the patch mechanism significantly worsened RMSE, MAE, MARD, and *R*^2^ in both full-period and postprandial evaluation. Removing seasonal decomposition also degraded performance, with consistent effects in the full-period setting and weaker deterioration in postprandial RMSE and *R*^2^. By contrast, the side branch had the smallest contribution. Most differences in the full-period setting were not significant, whereas in the postprandial setting, its removal led to a significant deterioration in RMSE and *R*^2^.

## Discussion

Our experiments demonstrated that LLM-based nutrient estimation yielded significant improvements in short-term glucose forecasting. LLMs were used to convert free-text dietary descriptions into structured nutritional features, which were integrated with historical CGM data in the proposed SPT forecasting model. This model achieved the lowest 3-h RMSE, reaching 26.963 mg/dl for full-period forecasting. Considering that prediction error generally increases as the forecasting horizon becomes longer, this result is competitive with a state-of-the-art CGM pre-training baseline (2-h RMSE = 26.88 mg/dl) [30]. Notably, the improvement was larger for postprandial glucose forecasting, reducing the 3-h postprandial RMSE by up to 1.967 mg/dl.

Heterogeneous feature integration remains challenging in continuous glucose forecasting. In our experiments, incorporating insulin medication, patient-level static features, or raw diet word embeddings degraded forecasting performance. Medication data, primarily insulin use, follow different distributions between the T1DM and T2DM cohorts [31], and this cohort-specific shift can confound model learning. Static clinical features were not associated with improved performance. These features are extremely sparse relative to CGM trajectories, and much of a patient’s underlying pancreatic function and metabolic state is already reflected in CGM patterns [32,33]. Therefore, adding static clinical labels may introduce redundant or inconsistent signals. Raw diet word embeddings learned from self-supervised pre-training prioritize linguistic context over nutritional information [16]. Nutritional content occupied only a small portion of the semantic space of these embeddings, which likely introduced irrelevant variation. Overall, these results suggest that LLM-derived nutrient estimation is more effective than high-dimensional semantic embeddings or static data for the continuous glycemic forecasting task.

We next linked forecasting gains to nutrient-estimation accuracy to identify which nutrients contribute most to improved prediction. We observed the strong associations between forecasting improvement and LLM estimation RMSE for carbohydrates, dietary fiber, and fat. This aligns with the physiology of glucose metabolism. Postprandial blood glucose is primarily influenced by carbohydrate intake, while fiber and fat consumption can mitigate the rate of glucose increase [34]. This physiological interpretability is an additional advantage of the LLM-based method.

The architectural ablation results help clarify why the proposed SPT performed well under the best-performing input setting. The patch-based encoding contributed the largest gain, temporal decomposition provided additional support, and the side branch played a smaller but still useful role, especially in the postprandial setting. The contribution of patch-based encoding was the most pronounced. In SPT, patching is applied after decomposition, so the transformer operates on short segments of the seasonal CGM component together with aligned patches of the auxiliary dynamic features. This design lets the model learn local sequence patterns and short-window cross-variable relationships more directly than point-wise tokenization. Temporal decomposition contributed a smaller but still meaningful benefit, particularly in the full-period evaluation, likely because it separates slowly varying background dynamics from faster meal-related fluctuations before sequence modeling. The side branch had the most modest effect, which is plausible because the main branch already receives the patched seasonal CGM signal together with the auxiliary dynamic inputs and therefore already captures most of the predictive information. Its contribution became more visible in the postprandial task, where an additional CGM-only pathway may provide cleaner support for modeling rapid glucose excursions.

Our comparison of 3 strategies for injecting nutrition information has practical implications for deploying LLM-assisted diet estimation in glucose-aware self-management systems. Combining the LLM with a nutrition database reduced computational cost without significantly affecting overall glucose-forecasting performance compared with using the LLM alone. The RAG-based strategy improved nutrient-estimation accuracy relative to approaches without RAG. However, the downstream gains in glucose forecasting were limited, and forecasting performance was similar between the RAG strategy and direct estimation. These results suggest that the added complexity of RAG should be weighed against its marginal benefit in practice. Finally, selecting an LLM for nutrient estimation should prioritize accurate estimation of carbohydrates, dietary fiber, and fat.

From a translational perspective, the proposed framework is compatible with a real-world deployment pipeline. Patients could record meals in free-text form through a mobile application or digital diary, consistent with prior studies demonstrating the feasibility of app-based dietary logging in diabetes self-management [35]. An LLM-based nutrient-estimation module could then convert these records into structured nutritional features, which can be integrated with CGM data to support personalized glucose monitoring and feedback, as shown in recent mobile health interventions combining diet tracking and CGM [36]. Importantly, this approach can substantially expand the range of foods that can be nutritionally annotated, overcoming the limitations of predefined food composition databases and enabling flexible handling of diverse real-world dietary inputs.

This translational perspective also helps position our study relative to recent CGM foundation-model work. These studies highlight the value of large-scale representation learning from CGM trajectories [30,33,37], and our work is complementary to that direction. In real-world settings, structured meal-related nutritional data are rarely available at scale and are difficult to collect manually. For example, the preprocessing described in GluFormer reduced the initial cohort from 10,844 participants to 5,875 participants after meal-related filtering [37]. Automated nutrient estimation from free-text meal records therefore provides a low-cost way to connect dietary records with forecasting models in a clinically interpretable form. Our findings on patch-based encoding and temporal decomposition may therefore also be informative for CGM foundation models that are currently built solely on transformer blocks.

We also note several limitations. First, the Shanghai cohorts were collected under free-living recordings, but the sample size and dietary variety remain limited. The imbalance between T1DM and T2DM may affect the generalizability of models, although we attempted to improve generalizability by pooled training. In addition, the lack of external validation limits the assessment of robustness across populations. The proposed feature fusion strategy may not fully capture patient-level information, and static features could become more useful with improved modeling and larger datasets [38,39]. Next, the real-world dietary records are prone to missing entries, typographical errors, and inaccurate portion sizes, placing an upper bound on the accuracy of any nutrient-estimation approach. Moreover, the nutrition database used to evaluate LLM-based estimation serves only as a reference rather than ground truth, and it does not support the evaluation of GI. This limitation may partly explain why improvements in nutrient-estimation accuracy do not translate monotonically into gains in glucose prediction performance. The correlation between nutrient-estimation accuracy and glucose-forecasting performance should be interpreted as an overall trend rather than a strict rank-preserving relationship. Variations in recipes and cooking methods also introduce unavoidable discrepancies between recorded and actual intakes. These factors collectively may account for the observation that direct estimation, database-assisted, and RAG strategies showed only small differences in forecasting performance. Finally, our RAG implementation was relatively simple, and more advanced RAG technologies and a larger nutrition database may yield further improvements [40].

Future work can extend this study in several directions. One direction is to combine additional meal-related signals, such as meal images, with LLM-based text parsing to reduce ambiguity and improve nutrient estimation [41]. Another direction is to refine the feature fusion framework to better preserve individual differences and move toward personalized digital twins for glucose management [42]. In addition, modeling the pharmacokinetics and dynamic effects of different glucose-lowering medications in T1DM and T2DM patients could further enhance predictive accuracy by capturing cohort-specific treatment dynamics. Finally, evaluating the method in larger cohorts with more diverse populations and dietary cultures will help assess generalizability and robustness.

## Conclusion

In conclusion, this study shows that LLM-based nutrient estimation can serve as a practical and physiologically interpretable bridge between unstructured free-text dietary records and short-term CGM forecasting. In the main experiments, structured nutrient features derived from LLMs outperformed raw semantic meal embeddings as dietary representations for glucose prediction, with the best average result obtained under direct nutrient estimation by DeepSeek-v3.1. The subsequent ablation analysis further showed that under the same DeepSeek-v3.1 setting without dynamic medication features, additional improvements were achieved, resulting in the best overall performance across all experiments. The patch-based encoding, temporal decomposition, and the side branch each contributed to the performance. These findings suggest that automated nutrient estimation can meaningfully enhance real-world glucose forecasting while also indicating that more robust integration of personalized patient information and broader external validation are still needed to support more generalizable deployment.

## Ethical Approval

The ShanghaiT1DM/T2DM datasets are publicly available data. Data collection was approved by the Ethics Committee of Shanghai Fourth People’s Hospital and Shanghai East Hospital affiliated to Tongji University, in accordance with the Declaration of Helsinki. Informed consent was obtained from all the patients. The present study involved a secondary analysis of de-identified data and did not require additional ethical approval.

## Data Availability

The ShanghaiT1DM/T2DM datasets can be accessed via the dataset providers’ publication [9]. The Chinese Food Composition database and dishes database will be available upon reasonable request [25].

## Supplementary Materials

Supplementary 1 Supplementary Methods S1.1 to S1.5 Figs. S1 and S2 Algorithm S1 Tables S1 to S4 Supplementary Results S2.1 to S2.3
