# Supplementary material for: Short-Term Continuous Glucose Forecasting with Large-Language-Model-Derived Nutrient Estimates from Real-World Chinese Dietary Records
Source: Health Data Sci. 2026 Oct 28;6:0471. doi: 10.34133/hds.0471 (PMC13714320; doi:10.34133/hds.0471)
Supplement: Supplementary file 1 [file hds.0471.f1.docx]

*Supplementary Information for*

**Short-Term Continuous Glucose Forecasting with Large Language Model-Derived Nutrient Estimates from Real-World Chinese Dietary Records**

Catalog

[S1. Supplementary Methods	2](#_Toc224691622)

[S1.1 Prompt template for nutrient estimation	2](#_Toc224691623)

**Figures S1.** The CoT-like prompt template for LLMs to estimate nutrients.

[S1.2 Prompt template for ingredient decomposition	3](#_Toc224691624)

**Figures S2.** The prompt template to decompose food ingredients.

[S1.3 RAG-based nutrient-estimation workflow	3](#_Toc224691625)

**Algorithm S1.** RAG-based nutrient-estimation workflow

[S1.4 Included features and preprocessing	5](#_Toc224691626)

**Table S1.** Included features and preprocessing

[S1.5 Supplementary cohort-specific analysis design	7](#_Toc224691627)

[S2. Supplementary Results	7](#_Toc224691628)

[S2.1 Detailed results of all experimental settings	7](#_Toc224691629)

**Table S2.** The average values and standard deviations of the evaluation metrics for all the experimental setups

[S2.2 Cohort-level differences in medication exposure	11](#_Toc224691630)

**Table S3.** Cohort-level differences in medication exposure between the Shanghai T1DM and Shanghai T2DM cohorts.

[S2.3 Cohort-specific mixed-versus-separate training analysis	11](#_Toc224691631)

**Table S4.** Cohort-specific comparison between mixed-cohort and cohort-specific training.

## S1. Supplementary Methods

### S1.1 Prompt template for nutrient estimation

We used a CoT-like prompt strategy to instruct the LLMs to estimate structured nutrient values from free-text Chinese dietary records. The prompt required the model to output nutrients in a structured JSON-like format and to explicitly record any non-standard serving units when present.

**Figures S1.** The CoT-like prompt template for LLMs to estimate nutrients. The placeholders {food_name} and {RAG_result} denote the input food description and the retrieved external evidence, respectively. Blue text beginning with # indicates explanatory text used for implementation purposes and is not included in the formal prompt delivered to the model.

### S1.2 Prompt template for ingredient decomposition

When direct food-level retrieval was insufficient, we prompted the LLM to decompose complex dishes into constituent ingredients before repeating database matching at the ingredient level.

**Figures S2.** The prompt template to decompose food ingredients. The placeholder {food_name} denotes the original Chinese food description to be decomposed. Blue text beginning with # indicates explanatory text for implementation and is not part of the formal prompt.

### S1.3 RAG-based nutrient-estimation workflow

**Notation.** Let $f$ denote a food item extracted from the dietary record, and let $D$ denote the local nutrition database queried by exact and fuzzy matching. The LLM used for context sufficiency judgment and ingredient decomposition is denoted by $M_{\mathrm{eval}}$, whereas the LLM used for final nutrient estimation is denoted by $M_{\mathrm{est}}$. The dish-level fuzzy-retrieval candidate set is denoted by $C_{\mathrm{dish}}$, the ingredient-level retrieved set by $C_{\mathrm{ing}}$, and the final retrieval context by $C_{\mathrm{final}}$. The final structured nutrient estimate is denoted by $y$.

The workflow can be summarized as follows:

- Dish-level preprocessing: segment $f$ with jieba search-mode tokenization to obtain searchable tokens
- Dish-level retrieval: retrieve and rank entries in $D$ whose food names contain the query tokens to form $C_{\mathrm{dish}}$
- Context sufficiency judgment: $(s,I)=M_{\mathrm{eval}}(f,C_{\mathrm{dish}})$
- Ingredient-level retrieval: if the dish-level context is insufficient, decompose $f$ into ingredients $I$, then for each ingredient first attempt exact matching and perform fuzzy retrieval only when exact matching fails, and aggregate the returned candidates into $C_{\mathrm{ing}}$
- Final context construction: $C_{\mathrm{final}}$ is set to $C_{\mathrm{dish}}$ if $s=true$, to $C_{\mathrm{ing}}$ if $s=false$ and ingredient-level matches are available, and to $⌀$ otherwise
- Final nutrient estimation: $y=M_{\mathrm{est}}(f,C_{\mathrm{final}})$

**Algorithm S1.** RAG-based nutrient-estimation workflow

**Input:** $f$ := food name from dietary record; $D$ := local nutrition database; $M_{\mathrm{eval}},M_{\mathrm{est}}$ := LLMs.
**Output:** $y$ := structured JSON nutrient estimate for $f$.

**Procedure:**

1. If $ExactMatch(f,D)$ exists, return the matched nutritional record.
2. Let $T:=JiebaSearchTokens(f)$.
3. Let $C_{\mathrm{dish}}:=FuzzyRetrieve(T,D)$.
4. Let $(s,I):=M_{\mathrm{eval}}(f,C_{\mathrm{dish}})$.
5. If $s=true$, let $C_{\mathrm{final}}:=C_{\mathrm{dish}}$.
6. Otherwise:
   6.1. Initialize $C_{\mathrm{ing}}:=⌀$.
   6.2. For each $i\in I$:
    6.2.1. Let $C_{i}:=TopK(RetrieveExactThenFuzzy(i,D),5)$.
    6.2.2. Update $C_{\mathrm{ing}}:=C_{\mathrm{ing}}\cup C_{i}$.
   6.3. Let $C_{\mathrm{final}}:=C_{\mathrm{ing}}$.
7. Let $P:=ConstructPrompt(f,C_{\mathrm{final}})$.
8. Let $y:=M_{\mathrm{est}}(P)$, and return $y$.

### S1.4 Included features and preprocessing

Table S1 summarizes all variables used in the forecasting experiments and the corresponding preprocessing operations.

Table S1. Included features and preprocessing

| **Feature Type** | **Features Included** | **Preprocessing** |
| --- | --- | --- |
| **Static Features** | 'Age (years)', 'Height (m)', 'Weight (kg)', 'Duration of diabetes (years)', 'Fasting Plasma Glucose (mg/dl)', '2-hour Postprandial Plasma Glucose (mg/dl)', 'Fasting C-peptide (nmol/L)', '2-hour Postprandial C-peptide (nmol/L)', 'Fasting Insulin (pmol/L)', '2-hour Postprandial Insulin (pmol/L)', 'HbA1c (mmol/mol)', 'Glycated Albumin (%)', 'Total Cholesterol (mmol/L)', 'Triglyceride (mmol/L)', 'High-Density Lipoprotein Cholesterol (mmol/L)', 'Low-Density Lipoprotein Cholesterol (mmol/L)', 'Creatinine (umol/L)', 'Estimated Glomerular Filtration Rate (ml/min/1.73m2) ', 'Uric Acid (mmol/L)', 'Blood Urea Nitrogen (mmol/L)', 'Comorbidities_Count', 'Gender (Female=1, Male=2)', 'Patient_Type' | Patient type was labeled based on diabetes classification. Comorbidities and complications were represented as a count of unique disease types. Categorical features were one-hot encoded. Continuous features were standardized by robust scalers based on training set statistics. Missing values were imputed using KNN imputation. |
| **Dynamic Features** | 'Recording_time', 'CBG', 'diet_word_embedding', 'diet_calories', 'diet_protein', 'diet_fat', 'diet_carbohydrates', 'diet_fiber', 'diet_GI', 'Insulin dose - s.c.', 'Non-insulin hypoglycemic agents', 'CSII - bolus insulin (Novolin R, IU)', 'CSII - basal insulin (Novolin R, IU / H)', 'Insulin dose - i.v.', | The dynamic features include recording time, medication information aligned with CGM, macro-nutrient estimation data, and dietary log text. The recording time of CGM was simplified to 0-24 hours. For any CGM data gaps of ≤ 1 hour (maximum four 4 points), linear interpolation was applied to impute missing values. Regarding the medication information, we used Boolean notation to indicate whether non-insulin hypoglycemic agents were used; we used regular expressions to handle the insulin dosage in the text, which is of a numerical type. The missing values of insulin dosages were regarded as 0. For dietary logs, 'unrecorded' entries were treated as missing data and imputed with the mean CGM value for the corresponding time interval; otherwise, no food intake was assumed (value of 0). Regular expressions were employed to parse Chinese food descriptions, extracting quantity, units, and food names. The food names were then deduplicated. We used LLMs to estimate the nutritional content of each 100g (or ml) of food, and then summed up the results weighted by the consumption amount. These food names were then converted to diet word embeddings using the pre-trained Chinese language model BGE-M3. The diet word embeddings at each moment were also weighted and fused based on the amount consumed. |

### S1.5 Supplementary cohort-specific analysis design

To further examine the rationale for training on the mixed cohort of T1DM and T2DM, we conducted a supplementary cohort-specific analysis. This analysis used the DeepSeek-v3.1 direct nutrient-estimation setting under the input configuration without dynamic medication information, because this configuration yielded the best performance in the ablation study. The T1DM-only and T2DM-only patient partitions were not independently re-sampled. Instead, they were derived from the corresponding cohort-specific subsets of the patient-level split used by the main pooled-cohort model. The hyperparameters used are the same as those in the main experiment. This analysis was conducted as a separate experimental batch, and therefore the results are different from those of the ablation experiments under the same settings.

## S2. Supplementary Results

### S2.1 Detailed results of all experimental settings

**Table S2.** The average values and standard deviations of the evaluation metrics for all the experimental setups

| Task | LLM | Diet word embedding | Static feature | RMSE | MAE | MARD | R2 | RMSE_std | MAE_std | MARD_std |
| --- | --- | --- | --- | --- | --- | --- | --- | --- | --- | --- |
| Postprandial | Baseline | FALSE | FALSE | 35.77 | 27.27 | 17.86 | 0.42 | 0.58 | 0.63 | 0.34 |
| Postprandial | w/o LLM | FALSE | FALSE | 36.63 | 27.77 | 18.35 | 0.39 | 0.75 | 0.63 | 0.35 |
| Postprandial | w/o LLM | FALSE | TRUE | 38.57 | 28.81 | 19.10 | 0.32 | 1.46 | 1.24 | 0.56 |
| Postprandial | w/o LLM | TRUE | FALSE | 38.64 | 29.59 | 19.76 | 0.32 | 0.83 | 0.62 | 0.71 |
| Postprandial | w/o LLM | TRUE | TRUE | 41.02 | 30.87 | 20.13 | 0.23 | 2.45 | 1.70 | 1.05 |
| Postprandial | Deepseekv3.1 | FALSE | FALSE | 34.67 | 26.15 | 17.58 | 0.45 | 0.40 | 0.48 | 0.24 |
| Postprandial | Deepseekv3.1 | FALSE | TRUE | 38.90 | 30.03 | 19.17 | 0.31 | 2.16 | 1.84 | 0.86 |
| Postprandial | Deepseekv3.1 | TRUE | FALSE | 37.15 | 28.50 | 20.03 | 0.37 | 1.12 | 0.93 | 1.12 |
| Postprandial | Deepseekv3.1 | TRUE | TRUE | 39.29 | 29.70 | 20.47 | 0.30 | 1.02 | 1.01 | 0.69 |
| Postprandial | Deepseekv3.1_db | FALSE | FALSE | 34.82 | 26.50 | 17.49 | 0.45 | 0.36 | 0.38 | 0.38 |
| Postprandial | Deepseekv3.1_db | FALSE | TRUE | 38.06 | 29.75 | 19.57 | 0.34 | 0.42 | 0.48 | 0.49 |
| Postprandial | Deepseekv3.1_db | TRUE | FALSE | 37.12 | 28.57 | 19.58 | 0.37 | 0.59 | 0.46 | 0.64 |
| Postprandial | Deepseekv3.1_db | TRUE | TRUE | 39.38 | 30.06 | 20.61 | 0.29 | 1.06 | 0.98 | 0.57 |
| Postprandial | Deepseekv3.1_w/o_Med | FALSE | FALSE | 34.48 | 26.15 | 17.46 | 0.46 | 0.24 | 0.31 | 0.21 |
| Postprandial | Deepseekv3.1_rag | FALSE | FALSE | 34.86 | 26.46 | 17.49 | 0.45 | 0.39 | 0.41 | 0.32 |
| Postprandial | Deepseekv3.1_rag | FALSE | TRUE | 38.60 | 29.52 | 19.54 | 0.32 | 1.00 | 1.00 | 0.73 |
| Postprandial | Deepseekv3.1_rag | TRUE | FALSE | 37.09 | 28.57 | 19.71 | 0.37 | 0.68 | 0.61 | 0.66 |
| Postprandial | Deepseekv3.1_rag | TRUE | TRUE | 39.85 | 30.13 | 20.54 | 0.28 | 1.08 | 1.02 | 1.04 |
| Postprandial | GLM4.5 | FALSE | FALSE | 35.14 | 26.74 | 17.61 | 0.44 | 0.50 | 0.46 | 0.19 |
| Postprandial | GLM4.5 | FALSE | TRUE | 38.38 | 29.50 | 19.16 | 0.33 | 1.59 | 1.58 | 0.64 |
| Postprandial | GLM4.5 | TRUE | FALSE | 36.98 | 28.40 | 19.85 | 0.38 | 0.86 | 0.81 | 0.99 |
| Postprandial | GLM4.5 | TRUE | TRUE | 40.56 | 30.50 | 20.44 | 0.25 | 1.86 | 1.35 | 0.76 |
| Postprandial | GLM4.5_db | FALSE | FALSE | 35.09 | 26.62 | 17.61 | 0.44 | 0.69 | 0.63 | 0.25 |
| Postprandial | GLM4.5_db | FALSE | TRUE | 38.14 | 29.23 | 19.19 | 0.34 | 1.71 | 1.72 | 0.46 |
| Postprandial | GLM4.5_db | TRUE | FALSE | 37.35 | 28.65 | 20.26 | 0.36 | 1.06 | 0.84 | 1.44 |
| Postprandial | GLM4.5_db | TRUE | TRUE | 39.14 | 29.53 | 20.24 | 0.30 | 1.50 | 1.08 | 0.96 |
| Postprandial | GPT5-mini | FALSE | FALSE | 34.98 | 26.53 | 17.58 | 0.44 | 0.49 | 0.46 | 0.35 |
| Postprandial | GPT5-mini | FALSE | TRUE | 38.11 | 29.46 | 18.92 | 0.34 | 1.56 | 1.29 | 0.47 |
| Postprandial | GPT5-mini | TRUE | FALSE | 36.90 | 28.31 | 19.36 | 0.38 | 0.46 | 0.42 | 0.64 |
| Postprandial | GPT5-mini | TRUE | TRUE | 39.82 | 30.22 | 20.64 | 0.28 | 1.34 | 1.42 | 0.64 |
| Postprandial | GPT5-mini_db | FALSE | FALSE | 34.85 | 26.43 | 17.61 | 0.45 | 0.40 | 0.49 | 0.36 |
| Postprandial | GPT5-mini_db | FALSE | TRUE | 37.63 | 28.90 | 18.81 | 0.36 | 1.08 | 0.82 | 0.65 |
| Postprandial | GPT5-mini_db | TRUE | FALSE | 37.28 | 28.63 | 19.93 | 0.37 | 0.60 | 0.66 | 0.69 |
| Postprandial | GPT5-mini_db | TRUE | TRUE | 39.23 | 29.73 | 20.14 | 0.30 | 1.39 | 1.40 | 0.64 |
| Postprandial | Llama3.1 | FALSE | FALSE | 35.12 | 26.59 | 17.63 | 0.44 | 0.43 | 0.50 | 0.38 |
| Postprandial | Llama3.1 | FALSE | TRUE | 38.35 | 29.37 | 19.30 | 0.33 | 1.21 | 1.08 | 0.41 |
| Postprandial | Llama3.1 | TRUE | FALSE | 36.83 | 28.27 | 19.53 | 0.38 | 0.48 | 0.58 | 0.52 |
| Postprandial | Llama3.1 | TRUE | TRUE | 39.26 | 29.76 | 20.26 | 0.30 | 1.38 | 1.10 | 0.86 |
| Postprandial | Llama3.1_db | FALSE | FALSE | 34.72 | 26.15 | 17.69 | 0.45 | 0.33 | 0.27 | 0.35 |
| Postprandial | Llama3.1_db | FALSE | TRUE | 38.39 | 29.57 | 19.29 | 0.33 | 1.24 | 1.26 | 0.51 |
| Postprandial | Llama3.1_db | TRUE | FALSE | 37.11 | 28.47 | 20.07 | 0.37 | 0.83 | 0.64 | 0.99 |
| Postprandial | Llama3.1_db | TRUE | TRUE | 38.61 | 29.51 | 19.83 | 0.32 | 0.82 | 0.78 | 0.46 |
| Postprandial | Qwen3 | FALSE | FALSE | 35.28 | 26.70 | 17.73 | 0.43 | 0.62 | 0.74 | 0.34 |
| Postprandial | Qwen3 | FALSE | TRUE | 38.25 | 29.21 | 19.47 | 0.33 | 1.27 | 1.30 | 0.43 |
| Postprandial | Qwen3 | TRUE | FALSE | 37.16 | 28.58 | 19.81 | 0.37 | 0.69 | 0.65 | 0.52 |
| Postprandial | Qwen3 | TRUE | TRUE | 39.52 | 29.86 | 20.46 | 0.29 | 1.41 | 1.08 | 0.76 |
| Postprandial | Qwen3_db | FALSE | FALSE | 34.85 | 26.33 | 17.61 | 0.45 | 0.42 | 0.36 | 0.29 |
| Postprandial | Qwen3_db | FALSE | TRUE | 38.06 | 29.24 | 19.15 | 0.34 | 0.91 | 0.96 | 0.51 |
| Postprandial | Qwen3_db | TRUE | FALSE | 36.92 | 28.40 | 19.74 | 0.38 | 0.86 | 0.74 | 0.94 |
| Postprandial | Qwen3_db | TRUE | TRUE | 39.27 | 29.69 | 20.08 | 0.30 | 0.97 | 0.98 | 0.52 |
| Full-period | Baseline | FALSE | FALSE | 27.40 | 18.81 | 14.19 | 0.62 | 0.16 | 0.16 | 0.17 |
| Full-period | w/o LLM | FALSE | FALSE | 27.45 | 18.80 | 14.16 | 0.62 | 0.21 | 0.16 | 0.22 |
| Full-period | w/o LLM | FALSE | TRUE | 32.97 | 21.84 | 15.56 | 0.45 | 1.95 | 1.12 | 0.61 |
| Full-period | w/o LLM | TRUE | FALSE | 27.76 | 18.83 | 14.47 | 0.61 | 0.28 | 0.18 | 0.29 |
| Full-period | w/o LLM | TRUE | TRUE | 29.19 | 19.57 | 14.77 | 0.57 | 0.98 | 0.60 | 0.36 |
| Full-period | Deepseekv3.1 | FALSE | FALSE | 27.11 | 18.58 | 14.02 | 0.63 | 0.21 | 0.22 | 0.19 |
| Full-period | Deepseekv3.1 | FALSE | TRUE | 30.84 | 20.66 | 15.32 | 0.52 | 1.01 | 0.56 | 0.45 |
| Full-period | Deepseekv3.1 | TRUE | FALSE | 27.46 | 18.68 | 14.29 | 0.62 | 0.40 | 0.26 | 0.62 |
| Full-period | Deepseekv3.1 | TRUE | TRUE | 29.78 | 19.95 | 14.73 | 0.55 | 1.68 | 1.05 | 0.77 |
| Full-period | Deepseekv3.1_db | FALSE | FALSE | 27.15 | 18.53 | 13.90 | 0.63 | 0.20 | 0.21 | 0.17 |
| Full-period | Deepseekv3.1_db | FALSE | TRUE | 30.89 | 20.68 | 15.15 | 0.52 | 0.98 | 0.74 | 0.44 |
| Full-period | Deepseekv3.1_db | TRUE | FALSE | 27.35 | 18.57 | 14.19 | 0.63 | 0.24 | 0.18 | 0.30 |
| Full-period | Deepseekv3.1_db | TRUE | TRUE | 28.93 | 19.48 | 14.47 | 0.58 | 0.59 | 0.44 | 0.57 |
| Full-period | Deepseekv3.1_w/o_Med | FALSE | FALSE | 26.96 | 18.47 | 13.83 | 0.64 | 0.15 | 0.12 | 0.11 |
| Full-period | Deepseekv3.1_rag | FALSE | FALSE | 27.03 | 18.50 | 13.84 | 0.63 | 0.16 | 0.12 | 0.12 |
| Full-period | Deepseekv3.1_rag | FALSE | TRUE | 30.91 | 20.75 | 15.38 | 0.52 | 0.93 | 0.67 | 0.78 |
| Full-period | Deepseekv3.1_rag | TRUE | FALSE | 27.48 | 18.67 | 14.26 | 0.62 | 0.36 | 0.34 | 0.28 |
| Full-period | Deepseekv3.1_rag | TRUE | TRUE | 29.14 | 19.51 | 14.74 | 0.57 | 1.23 | 0.74 | 0.54 |
| Full-period | GLM4.5 | FALSE | FALSE | 27.08 | 18.53 | 13.93 | 0.63 | 0.22 | 0.27 | 0.31 |
| Full-period | GLM4.5 | FALSE | TRUE | 31.82 | 21.27 | 16.03 | 0.49 | 1.74 | 1.13 | 0.89 |
| Full-period | GLM4.5 | TRUE | FALSE | 27.39 | 18.58 | 14.34 | 0.62 | 0.24 | 0.13 | 0.27 |
| Full-period | GLM4.5 | TRUE | TRUE | 29.20 | 19.59 | 14.59 | 0.57 | 1.63 | 0.92 | 0.36 |
| Full-period | GLM4.5_db | FALSE | FALSE | 27.12 | 18.56 | 13.94 | 0.63 | 0.17 | 0.14 | 0.15 |
| Full-period | GLM4.5_db | FALSE | TRUE | 30.66 | 20.61 | 15.38 | 0.53 | 1.25 | 0.78 | 0.69 |
| Full-period | GLM4.5_db | TRUE | FALSE | 27.41 | 18.64 | 14.16 | 0.62 | 0.32 | 0.27 | 0.29 |
| Full-period | GLM4.5_db | TRUE | TRUE | 29.03 | 19.53 | 14.89 | 0.58 | 1.02 | 0.71 | 0.79 |
| Full-period | GPT5-mini | FALSE | FALSE | 27.23 | 18.55 | 14.12 | 0.63 | 0.23 | 0.18 | 0.17 |
| Full-period | GPT5-mini | FALSE | TRUE | 31.49 | 21.05 | 15.59 | 0.50 | 0.92 | 0.55 | 0.63 |
| Full-period | GPT5-mini | TRUE | FALSE | 27.27 | 18.53 | 14.14 | 0.63 | 0.34 | 0.20 | 0.26 |
| Full-period | GPT5-mini | TRUE | TRUE | 28.85 | 19.42 | 14.45 | 0.58 | 0.90 | 0.56 | 0.39 |
| Full-period | GPT5-mini_db | FALSE | FALSE | 27.08 | 18.49 | 13.98 | 0.63 | 0.21 | 0.16 | 0.20 |
| Full-period | GPT5-mini_db | FALSE | TRUE | 30.06 | 20.40 | 15.33 | 0.55 | 0.68 | 0.55 | 0.72 |
| Full-period | GPT5-mini_db | TRUE | FALSE | 27.48 | 18.71 | 14.47 | 0.62 | 0.29 | 0.25 | 0.42 |
| Full-period | GPT5-mini_db | TRUE | TRUE | 29.34 | 19.76 | 14.80 | 0.57 | 0.87 | 0.67 | 0.78 |
| Full-period | Llama3.1 | FALSE | FALSE | 27.12 | 18.49 | 13.92 | 0.63 | 0.21 | 0.12 | 0.22 |
| Full-period | Llama3.1 | FALSE | TRUE | 31.61 | 21.17 | 15.50 | 0.50 | 1.04 | 0.69 | 0.53 |
| Full-period | Llama3.1 | TRUE | FALSE | 27.52 | 18.72 | 14.40 | 0.62 | 0.44 | 0.29 | 0.54 |
| Full-period | Llama3.1 | TRUE | TRUE | 28.88 | 19.39 | 14.74 | 0.58 | 1.16 | 0.67 | 0.62 |
| Full-period | Llama3.1_db | FALSE | FALSE | 27.16 | 18.64 | 13.94 | 0.63 | 0.13 | 0.17 | 0.18 |
| Full-period | Llama3.1_db | FALSE | TRUE | 31.28 | 21.06 | 15.86 | 0.51 | 0.99 | 0.59 | 0.92 |
| Full-period | Llama3.1_db | TRUE | FALSE | 27.65 | 18.74 | 14.48 | 0.62 | 0.27 | 0.18 | 0.19 |
| Full-period | Llama3.1_db | TRUE | TRUE | 28.86 | 19.38 | 14.46 | 0.58 | 0.97 | 0.62 | 0.25 |
| Full-period | Qwen3 | FALSE | FALSE | 27.24 | 18.65 | 13.94 | 0.63 | 0.17 | 0.16 | 0.15 |
| Full-period | Qwen3 | FALSE | TRUE | 31.89 | 21.32 | 15.41 | 0.49 | 1.28 | 0.79 | 0.63 |
| Full-period | Qwen3 | TRUE | FALSE | 27.42 | 18.57 | 14.33 | 0.62 | 0.30 | 0.17 | 0.37 |
| Full-period | Qwen3 | TRUE | TRUE | 29.00 | 19.48 | 14.41 | 0.58 | 0.74 | 0.50 | 0.24 |
| Full-period | Qwen3_db | FALSE | FALSE | 27.10 | 18.51 | 13.92 | 0.63 | 0.20 | 0.16 | 0.12 |
| Full-period | Qwen3_db | FALSE | TRUE | 31.13 | 20.92 | 15.42 | 0.51 | 0.81 | 0.43 | 0.43 |
| Full-period | Qwen3_db | TRUE | FALSE | 27.47 | 18.64 | 14.30 | 0.62 | 0.29 | 0.21 | 0.39 |
| Full-period | Qwen3_db | TRUE | TRUE | 28.65 | 19.21 | 14.44 | 0.59 | 0.81 | 0.55 | 0.34 |

Note: The “w/o” notes “without”. The “db” suffix indicates the method of combining with the nutrition database, the “rag” suffix indicates that the RAG technique is used, and the “w/o_Med” suffix indicates that no medication information is used. Bold marks the best-performing model for each task, and underlining marks the second-best in the task. RMSE, MAE, and MARD are measured in units of mg/dL. The R^2^ value has no unit.

### S2.2 Cohort-level differences in medication exposure

Table S3 summarizes the medication-exposure differences between the Shanghai T1DM and Shanghai T2DM cohorts to support the interpretation of the medication-related ablation results.

**Table S3.** Cohort-level differences in medication exposure between the Shanghai T1DM and Shanghai T2DM cohorts.

| Variable | Shanghai T1DM | Shanghai T2DM | Fisher's exact test |
| --- | --- | --- | --- |
| Any insulin exposure | 12/12 (100.0%) | 60/100 (60.0%) | P = 0.0040 |
| Non-insulin hypoglycemic agents | 6/12 (50.0%) | 84/100 (84.0%) | P = 0.0124 |
| CSII bolus insulin | 9/12 (75.0%) | 34/100 (34.0%) | P = 0.0098 |
| CSII basal insulin | 9/12 (75.0%) | 34/100 (34.0%) | P = 0.0098 |
| Intravenous insulin | 5/12 (41.7%) | 6/100 (6.0%) | P = 0.0019 |
| Subcutaneous insulin | 10/12 (83.3%) | 49/100 (49.0%) | P = 0.0318 |

Note: CSII for continuous subcutaneous insulin infusion.

### S2.3 Cohort-specific pooled-versus-separate training analysis

Table S4 summarizes the comparison between pooled-cohort and cohort-specific training under the DeepSeek-v3.1 direct-estimation setting without dynamic medication information. In T1DM, pooled-cohort training substantially reduced errors (full-period RMSE: 30.73 vs. 39.19 mg/dL; postprandial RMSE: 38.43 vs. 49.09 mg/dL) with consistent gains in MAE, MARD, and R². In T2DM, both training strategies performed similarly, indicating that pooled-cohort training primarily benefits the smaller T1DM cohort while maintaining performance in the larger T2DM cohort.

**Table S4.** Cohort-specific comparison between pooled-cohort and separate-cohort training.

| **Task** | **Test cohort** | **RMSE (Mixed / Cohort-specific, mg/dL) ↓** | **MAE (Mixed / Cohort-specific, mg/dL) ↓** | **MARD (Mixed / Cohort-specific) ↓** | **R² (Mixed / Cohort-specific) ↑** |
| --- | --- | --- | --- | --- | --- |
| Full-period | T1DM | 30.73 / 39.19 | 20.88 / 27.32 | 17.62 / 21.64 | 0.69 / 0.49 |
|  | T2DM | 26.68 / 26.69 | 18.26 / 18.16 | 13.62 / 13.73 | 0.63 / 0.63 |
| Postprandial | T1DM | 38.43 / 49.09 | 29.14 / 40.12 | 26.37 / 32.73 | 0.16 / -0.37 |
|  | T2DM | 34.39 / 34.20 | 26.22 / 26.30 | 16.69 / 16.39 | 0.43 / 0.44 |
